# Mixed Wastewater Coupled with CO_2_ for Microalgae Culturing and Nutrient Removal

**DOI:** 10.1371/journal.pone.0139117

**Published:** 2015-09-29

**Authors:** Lili Yao, Jianye Shi, Xiaoling Miao

**Affiliations:** 1 State Key Laboratory of Microbial Metabolism and School of Life Sciences & Biotechnology, Shanghai Jiao Tong University, 800 Dongchuan Road, Shanghai 200240, China; 2 Biomass Energy Research Center, Shanghai Jiao Tong University, Shanghai 200240, China; CAS, CHINA

## Abstract

Biomass, nutrient removal capacity, lipid productivity and morphological changes of *Chlorella sorokiniana* and *Desmodesmus communis* were investigated in mixed wastewaters with different CO_2_ concentrations. Under optimal condition, which was 1:3 ratio of swine wastewater to second treated municipal wastewater with 5% CO_2_, the maximum biomass concentrations were 1.22 g L^-1^ and 0.84 g L^-1^ for *C*. *sorokiniana* and *D*. *communis*, respectively. Almost all of the ammonia and phosphorus were removed, the removal rates of total nitrogen were 88.05% for *C*. *sorokiniana* and 83.18% for *D*. *communis*. Lipid content reached 17.04% for *C*. *sorokiniana* and 20.37% for *D*. *communis* after 10 days culture. CO_2_ aeration increased intracellular particle numbers of both microalgae and made *D*. *communis* tend to be solitary. The research suggested the aeration of CO_2_ improve the tolerance of microalgae to high concentration of NH_4_-N, and nutrient excess stress could induce lipid accumulation of microalgae.

## Introduction

Concerns about the depletion of petroleum resources reserves [1] coupled with the rise of the global energy demand, and an increasing awareness of the environmental impact of associated CO_2_ emissions, have made the development of renewable and environmentally friendly energy sources necessary [2]. In this sense, biodiesel which has properties similar to fossil-fuels production from photosynthetic microorganisms has been recognized as reliable and renewable energy sources for the steady supply of energy. Many studies have demonstrated that microalgae were superior to other raw materials for the production of biodiesel [3–4]. However, the development of microalgae-based biodiesel still faces many challenges. One of the most critical challenges is to establish economical means of supplying water and nutrients for cultivation since microalgae require a huge volume of medium for mass scale growth, which leading to major operating costs associated with the nutritional supply [5]. Meanwhile, the reclamation of wastewater is of pivotal importance to achieving sustainability in our society at the global level. Fortunately, in addition to their high biomass and lipid productivities, some microalgae strains also have potential environmental benefits, such as mitigation of CO_2_ through photosynthesis [6] and bioremediating wastewater by removing large amounts of nutrients and heavy metals [7]. Therefore, an algae-based wastewater and CO_2_ treatment system may be the key to solve both problems.

Swine wastewater (SW) often contained high concentrations of nitrogen and phosphorus, and it needed dilution with fresh water before use to achieve a high yield of biomass [5, 8] as well as to increase the transmission of light in microalgal cultures. Meanwhile, secondary treated municipal wastewater (STMW) supported the microalgal growth and lipid production, but the low concentrations of nutritional constituents result in a low biomass yield [9]. Hence, swine wastewater and municipal wastewater mixture might have great potential to provide good substrates for microalgal growth and get a high yield of lipid without the need for dilution with fresh water or nutrients supplementation. Some microalgae also show better growth potential under high CO_2_ concentrations [10–11] and have potential of mitigating flue gas CO_2_ through photosynthesis [6]. Thus, coupling mixed wastewater with CO_2_ might be an efficient mode for microalgae to produce biodiesel feedstock along with wastewater and CO_2_ treatment.

In this study, different culture systems with a series of wastewater mixed from SW and STMW, together with different CO_2_ concentrations were established. The biomass production, nutrient removal capacity, lipid yield and morphological change of two selected microalgae strains *Chlorella sorokiniana* and *Desmodesmus communis* in these different culture systems were investigated. The better culture conditions were proposed.

## Materials and Methods

### Pretreatment and preparation of different wastewater media

To collect the SW and STMW, we obtained permission from a key piggery, Minhang Breeding Stock Farm (Shanghai, China), and the Minhang Municipal Wastewater Treatment Plant.

SW was generated during the barn flush operations and then was passed through a bar screen and an inclined screen to achieve preliminary solid-liquid separation. After these processes, the wastewater was discharged to a primary sedimentation pond to precipitate the remaining solids, before slowly flowing into the storage pond from which SW was obtained. STMW used throughout the experiments was collected from the secondary treatment pond. Both SW and STMW samples were immediately filtered using microfilters (GB/T1914-93) to remove suspended particles after sampling and then were stored in a refrigerator at -20°C to avoid variation in the wastewater composition.

The different wastewater media were prepared using different proportions of two types of wastewater. The different proportions (v:v) of SW to STMW were as follows: SW 4:0 STMW (4:0); SW 3:1 STMW (3:1); SW 2:2 STMW (2:2); SW 1:3 STMW (1:3); an SW 0:4 STMW (0:4). The media were characterized in terms of ammonium nitrogen (NH_4_^+^-N), nitrate (NO_3_^-^-N), total nitrogen (TN), total phosphorus (TP) and pH, and the results are shown in Table 1. For ease of Compared with the modified BG-11 medium [12], we considered media 4:0 and 3:1 as media containing high levels of nitrogen and phosphorus. Correspondingly, media 2:2 and 1:3 were considered to have intermediate levels of nitrogen and phosphorus, and medium 0:4 was considered to have low levels of nitrogen and phosphorus.

**Table 1:** Characters of total nitrogen (TN), ammonium nitrogen (NH4^+^-N), nitrate (NO3^-^-N), total phosphorus (TP) and pH in BG-11 and different wastewater media.

Medium(SW: STMW)	TN(mg L^-1^)	NH_4_^+^-N(mg L^-1^)	NO_3_^-^-N(mg L^-1^)	TP(mg L^-1^)	pH
BG-11	247.059	0.000	247.059	5.345	8.02
0:4	39.851±0.495	9.189±0.105	21.042±0.059	0.424±0.002	7.39
1:3	188.611±1.003	104.392±0.860	61.026±0.919	15.768±0.204	8.04
2:2	337.320±4.869	199.963±0.368	104.905±1.503	30.877±0.034	8.12
3:1	477.179±8.283	293.032±1.942	146.531±1.771	46.094±0.007	8.16
4:0	632.986±2.643	387.226±2.662	188.496±3.163	61.534±0.568	8.16

SW**: s**wine wastewater, STMW: secondary treated municipal wastewater.

### Microalgae cultivation

The two microalgae *Chlorella sorokiniana* and *Desmodesmus communis* were screened from acid swege and selected based on the tolerance and performance under high concentration of CO_2_ by the Biomass Energy Research Center of Shanghai Jiao Tong University, China. These two strains could achieve relatively high biomass concentration when cultivated in wastewater medium with high level of CO_2_, and have potential to form the wastewater and CO_2_ treatment system. They were preserved in the modified BG-11 medium containing (g L^-1^) NaNO_3_, 1.5; K_2_HPO_4_, 0.03; MgSO_4_•2H_2_O, 0.075; CaCl_2_•2H_2_O, 0.036; citric acid, 0.006; ferric ammonium citrate, 0.006; EDTA, 0.001; Na_2_CO_3_, 0.020 and 1 mL of micronutrient solution containing (g L^-1^) H_3_BO_3_, 2.86; MnCl_2_•4H_2_O, 1.81; ZnSO_4_•7H_2_O, 0.222; NaMoO_4_•5H_2_O, 0.390; CuSO_4_•5H_2_O, 0.0790; Co(NO_3_)_2_•6H_2_O, 0.0494 [12]. The two strains were individually cultured in 250 mL Erlenmeyer flasks containing 120 mL sterilized modified BG-11 before inoculation to formulated wastewater media.

In different cultivation experiments, *C*. *sorokiniana* and *D*. *communis* were cultivated in 1 L Erlenmeyer flask (20 cm length, 10 cm diameter) with 600 mL working volume of different wastewater media at 28±2°C under 126 μmol m^-2^ s^-1^ light intensity on a light/dark cycle of 12 h/12 h for 10 days. The light intensity was measured by a light meter. A gas distributor provided with different flow rates of CO_2_ mixed with ambient air was used to prepare CO_2_ concentrations of 0.03% (air), 5% and 10%. Cultures were aerated continuously with CO_2_-enriched air via bubbling from the bottom of modified Erlenmeyer flask with an aeration rate of 0.2 vvm (volume gas per volume media per minute). The control (wastewater without algal inoculums) was conducted under the same conditions.

### Morphological analysis

The images of *C*. *sorokiniana* and *D*. *communis* were observed every other day during the cultivation by an optical microscope (OLYMPUS, CX41, magnification up to 1,000X), and the morphology was documented using a Mshot Digital Imaging System (MC50, Mshot, China).

### Cell growth measurement

Biomass concentrations (x, g L^-1^) were determined directly by dry cell weight. 10 mL sample was taken from culture to measure the dry cell weight daily. Microalgae were harvested by centrifugation (5804R, Eppendorf, Germany) at 8000 rpm for 10 min and washed twice with distilled water. The pellet was lyophilized drying in a freeze drier (FD-1-50, Boyikang, China) for dry weight measurement.

The biomass productivity P (g L^-1^ d^-1^) and specific growth rate μ (d^-1^) were calculated according to the following Eqs (1) and (2):

P=(X1−X0)(t1−t0) (1)

μ=ln(X1X0)(t1−t0) (2)

where X_1_ and X_0_ were the dry cell weight concentration (g L^-1^) at time t_1_ and t_0_, respectively.

### Lipid extraction and quantification

The chloroform/methanol method was used for total lipid content measurement [13]. Dry microalgal cells (0.2 g) added with 6 mL distilled water and ultrasonicated by a sonicator (JY, 92-II, China) for 8 min, then mixed with solvent of chloroform:methanol (2:1, V/V) and left over night, the cell debris were removed by centrifugation at 8000 rpm for 10 min, the chloroform layer was extracted and transferred to a new screw-cap tube. Chloroform was added again to give a constant solvent ratio, and the residual cell debris were extracted three times by above solvent extraction procedure to ensure that lipids were almost extracted. The chloroform layer in the new screw-cap tube was washed with the same volume of 0.1% NaCl solution to wash out soluble impurities and the purified chloroform layer was evaporated to a constant weight in a fuming hood under vacuum at 60°C. The total lipid content (Lc, % of biomass dry weight) was calculated using the following equation:

Lc=(m2-m0)m1×100% (3)

where m_1_ was the weight of the dry microalgal cells, m_0_ was the weight of the empty new screw-cap tube, m_2_ was the weight of the new screw-cap tube with the dried lipids.

### Nitrate and phosphate concentration analysis

The nutrients (NH_4_^+^-N, TN and TP) uptake rate was measured every other day during the cultivation. A 10 mL liquid culture sample was centrifuged at 8000 rpm for 10 min, and the supernatant was filtered through a 0.45 μm syringe filter. total nitrogen (TN) and ammonium nitrogen (NH_4_^+^-N) were measured using an automatic chemistry analyzer (Smartchem 200, Alliance, France), total phosphorus (TP) in the medium was measured using acid potassium persulfate digestion by molybdenum antimony-colorimetric method [14].

## Results and Discussion

### Growth of microalgae in different wastewater media and the CO_2_ concentration

**Fig 1:**
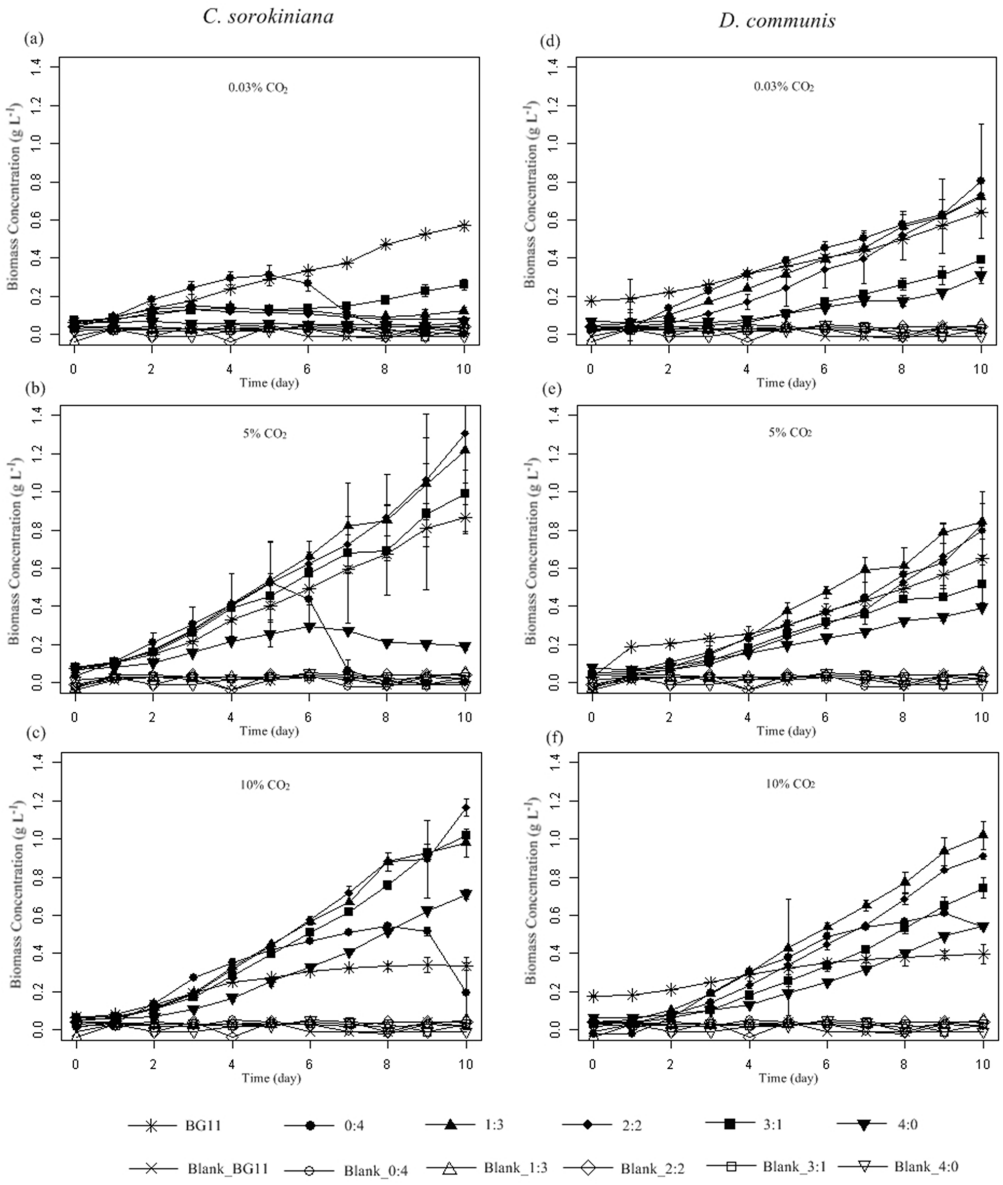
Biomass concentrations of *Chlorella sorokiniana* (a,b,c) and *Desmodesmus communis* (d,e,f) in different media under 0.03%, 5% and 10% CO_2_ concentrations, respectively. Media without microalgae inoculation was marked as Blank, Error bars represent ± SD of three replicates.

As previous studies have reported, the concentrations of nitrogen and phosphorus from media significantly affected microalgae growth [15–17]. The growth of *C*. *sorokiniana* and *D*. *communis* cultured in different mixing ratios of wastewater under different CO_2_ concentrations are shown in Fig 1. Under 0.03% CO_2_ (without extra CO_2_ aeration), *C*. *sorokiniana* showed better growth in modified BG-11 and medium 0:4 (Fig 1a). The maximum biomass concentrations were 0.57 g L^-1^ and 0.31 g L^-1^ in BG-11 and medium 0:4 after 10 days cultivation, respectively (Table 2). The growth of *C*. *sorokiniana* in media 1:3, 2:2, 3:1 and 4:0 were nearly inhibited under 0.03% CO_2_ (Fig 1a, Table 2). Ruangsomboon reported, within the range of 22 to 444 mg L^-1^, that the increase in the phosphorus concentration was beneficial to microalgal growth [16]. Because the concentration of phosphorus was within this range (Table 1), we inferred that NH_4_^+^-N might be the main factor inhibiting the growth of C. *sorokinian*a. It is well known that ammonia nitrogen above a particular concentration, which is microalgal species and culture pH dependent, would inhibit microalgal growth and reduce the utilization of wastewaters [18–19]. The main mechanism by which ammonia inhibits microalgae is by poisoning their photosynthetic system [20–21].

**Table 2:** The maximum biomass concentration (X_max_), maximum biomass productivity (P_max_) and maximum specific growth rate (μ_max_) of *Chlorella sorokiniana* and *Desmodesmus communis* cultivated in different media under 0.03%, 5% and 10% CO_2_ concentrations, respectively.

	Medium(SW: STMW)	0.03% CO_2_			5% CO_2_			10% CO_2_		
		X_max_ (g L^-1^)	P_max_ (g L^-1^ d^-1^)	μ_max_ (d^-1^)	X_max_ (g L^-1^)	P_max_ (g L^-1^ d^-1^)	μ_max_ (d^-1^)	X_max_ (g L^-1^)	P_max_ (g L^-1^ d^-1^)	μ_max_ (d^-1^)
*C*. *sorokiniana*	BG-11	0.57±0.01	0.099±0.002	0.465±0.012	0.86±0.07	0.136±0.004	0.425±0.064	0.34±0.04	0.079±0.001	0.519±0.002
	0:4	0.31±0.05	0.088±0.016	0.663±0.016	0.53±0.01	0.116±0.013	0.637±0.067	0.54±0.01	0.133±0.008	1.305±0.016
	1:3	0.15±0.00	0.044±0.006	0.402±0.019	1.22±0.05	0.193±0.023	0.519±0.017	0.98±0.07	0.211±0.010	0.554±0.003
	2:2	0.13±0.00	0.037±0.011	0.400±0.050	1.31±0.02	0.247±0.005	0.511±0.021	1.16±0.05	0.271±0.020	0.586±0.006
	3:1	0.26±0.03	0.050±0.001	0.273±0.029	0.99±0.06	0.193±0.008	0.495±0.005	1.02±0.00	0.168±0.003	0.514±0.015
	4:0	0.07±0.01	0.020±0.003	0.099±0.002	0.30±0.01	0.080±0.001	0.397±0.017	0.71±0.03	0.109±0.000	0.434±0.012
*D*. *communis*	BG-11	0.64±0.01	0.071±0.007	0.208±0.003	0.65±0.07	0.155±0.002	1.779±0.002	0.40±0.05	0.181±0.001	0.163±0.000
	0:4	0.80±0.05	0.172±0.025	1.514±0.038	0.83±0.06	0.205±0.012	0.408±0.051	0.61±0.01	0.128±0.008	1.110±0.035
	1:3	0.72±0.01	0.113±0.006	0.577±0.057	0.84±0.05	0.174±0.032	0.629±0.053	1.02±0.07	0.163±0.021	0.724±0.026
	2:2	0.73±0.01	0.120±0.006	0.636±0.058	0.80±0.03	0.148±0.005	0.554±0.009	0.91±0.01	0.151±0.022	0.818±0.024
	3:1	0.39±0.02	0.078±0.015	0.573±0.015	0.52±0.02	0.078±0.013	0.655±0.006	0.74±0.05	0.117±0.014	0.628±0.033
	4:0	0.31±0.04	0.090±0.003	0.343±0.004	0.39±0.02	0.055±0.001	0.323±0.035	0.54±0.01	0.086±0.011	0.367±0.001

The growth of *C*. *sorokiniana* in media containing SW was significantly increased when extra CO_2_ was aerated (Fig 1b and 1c). Under 5% CO_2_, *C*. *sorokiniana* achieved the highest maximum biomass concentration (1.31 g L^-1^) and maximum biomass productivity (0.247 g L^-1^ d^-1^) in medium 2:2, followed by 1.22 g L^-1^ and 0.193 g L^-1^ d^-1^ in medium 1:3 (Table 2). When 10% CO_2_ was aerated, the highest maximum biomass concentration (1.16 g L^-1^) and maximum biomass productivity (0.271 g L^-1^ d^-1^) were also achieved in medium 2:2 (Table 2). Additionally, when cultivated in 1:3 and 2:2 and modified BG-11, *C*. *sorokiniana* achieved higher biomass concentration under 5% CO_2_ than under 10% CO_2_, a finding that was consistent with previous studies that the growth of microalgae would be inhibited if the aerated CO_2_ was above a particular concentration [11, 21]. However, *C*. *sorokiniana* in media 3:1 and 4:0, which contain a relatively high concentration of nitrogen and phosphorus, grew better under 10% CO_2_ than 5% CO_2_ (Fig 1b, 1c and Table 2). These results suggested that the aeration of CO_2_ could improve the tolerance of *C*. *sorokiniana* with a high initial concentration of NH_4_^+^-N, and medium with a higher NH_4_^+^-N concentration might prefer a higher concentration of CO_2_ aeration. The aeration of CO_2_ probably changed the pH of the medium, leading to the change in the uptake pattern for nutrimental elements [22–23].

Although growing relatively better than *C*. *sorokiniana* at 0.03% CO_2_, *D*. *communis* showed a similar trend when extra CO_2_ was aerated (Fig 1d, 1e and 1f). *D*. *communis* obtained the highest maximum biomass concentrations of 0.84 g L^-1^ and 1.02 g L^-1^ in medium 1:3 with 5% and 10% CO_2_, respectively (Table 2). It was interesting to note that *D*. *communis* grew better under 5% CO_2_ than under 10% CO_2_ when it was cultivated in medium 0:4 and modified BG-11 (Fig 1e, 1f and Table 2). However, when *D*. *communis* was grown in other media, it grew better under 10% CO_2_ than 5% CO_2_ (Fig 1d, 1e, 1f and Table 2). These results suggested that the optimal CO_2_ concentration for microalgal growth was medium and strain dependent. Finding the proper wastewater composition coupled with the optimal CO_2_ concentration is a feasible way to promote the production of microalgal biomass.

### Nutrient removal during cultivation

**Fig 2:**
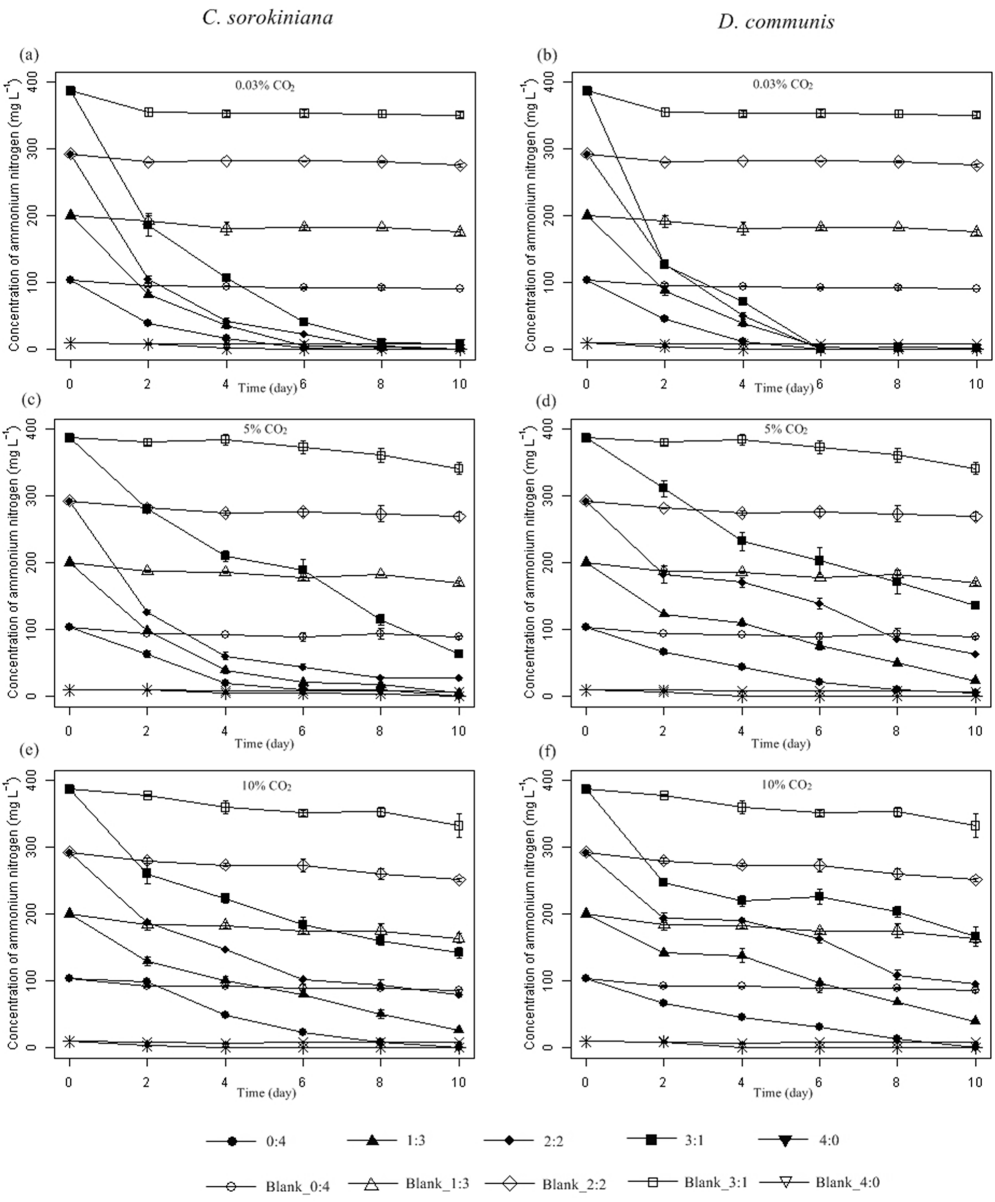
Time course of ammonium nitrogen (NH4^+^-N) evolution for *Chlorella sorokiniana* and *Desmodesmus communis* cultivated in different media under 0.03% (a and b), 5% (c and d) and 10% (e and f) CO_2_ concentrations, respectively. Media without microalgae inoculation was marked as Blank, Error bars represent ±SD of three replicates.

**Fig 3:**
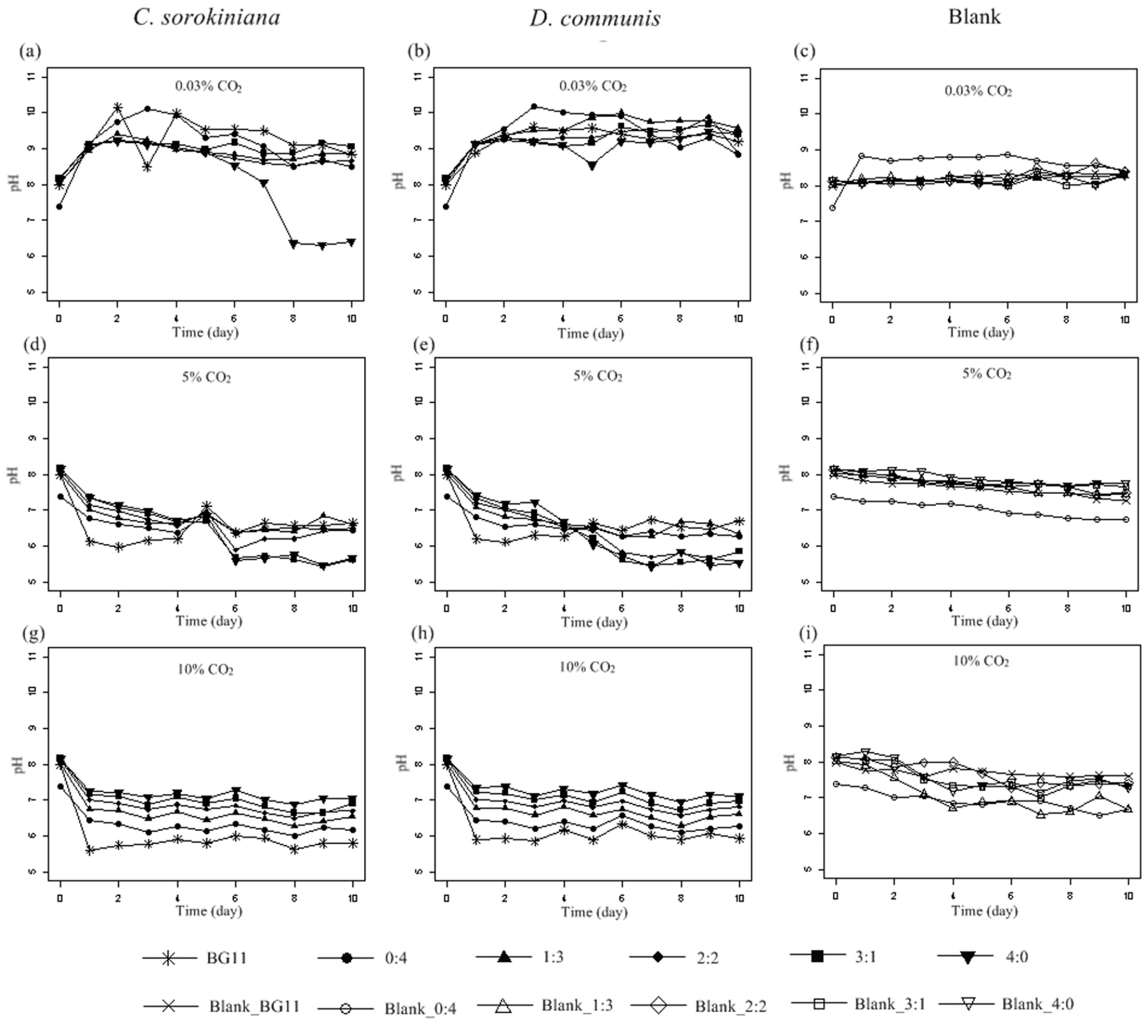
Time course of pH evolution for *Chlorella sorokiniana*, *Desmodesmus communis* and control (Blank) cultivated in different media under 0.03% (a, b and c), 5% (d, e and f) and 10% (g, h and i) CO_2_ concentrations, respectively. Error bars represent ± SD of three replicates.

Microalgae-based nutrient removal in wastewater is a much accepted concept worldwide. Nutrient-rich wastewater has been considered to be more appropriate for microalgal growth because it enables an increment in biomass concentration along with nutrient removal [24]. As shown in Table 1, medium 4:0 (SW:STMW) contained the highest levels of TN, NH_4_^+^-N and TP, while medium 0:4 (SW:STMW) had the lowest content of these nutrients. The analysis of NH_4_^+^-N showed that nearly all ammonia in the different media was removed by *C*. *sorokiniana* and *D*. *communis* under 0.03% CO_2_ (Fig 2a, 2b and Table 3). A similar phenomenon was also found by other scientists [22–23, 25–26]. The removal of ammonia was not only due to its up-take by *C*. *sorokiniana* and *D*. *communis* but also due to stripping and loss to the atmosphere. It has been noted that ammonia stripping and loss to the atmosphere may be the most important mechanisms of ammonia removal when microalgae or cyanobacteria are used for nutrient removal from wastewater [27]. Previous researchers have found that when media contained a high initial concentration of ammonia, the intensified growth inhibition would cause a decrease in ammonia uptake; consequently, ammonia was more susceptible to be stripped and lost, particularly in alkaline medium [26]. In the present study, a net increase in pH values without extra CO_2_ aeration was observed (Fig 3a and 3b), increasing the removal rate of ammonia.

**Table 3:** The removal rate of total nitrogen (TN), ammonium nitrogen (NH4^+^-N) and total phosphorus (TP) by *C*. *sorokiniana* and *D*. *communis* cultivated in different media under 0.03%, 5% and 10% CO_2_ concentrations, respectively. Media without microalgae inoculation was marked as Blank.

	Medium(SW: STMW)	Removal rate (%)								
		0.03% CO_2_			5% CO_2_			10% CO_2_		
		TN	NH_4_-N	TP	TN	NH_4_-N	TP	TN	NH_4_-N	TP
*C*. *sorokiniana*	0:4	50.59±3.95	100.00±0.00	100.00±0.00	50.38±1.79	93.60±5.75	89.99±0.56	55.71±5.65	97.50±1.57	95.00±7.06
	1:3	74.34±1.82	99.19±0.44	90.79±0.81	88.05±1.62	100.00±0.00	99.63±0.22	87.22±1.58	99.19±0.56	99.73±0.00
	2:2	74.63±7.72	99.74±0.12	80.90±0.90	63.90±1.24	97.51±1.84	93.02±0.10	75.61±0.37	87.20±3.90	91.43±0.10
	3:1	74.48±0.74	99.88±0.13	90.80±0.15	57.74±3.39	90.63±4.25	76.15±12.83	77.64±0.84	73.05±4.36	83.35±0.00
	4:0	40.43±1.05	98.04±2.14	90.64±3.32	56.34±1.16	83.60±4.57	75.98±5.86	74.45±9.68	63.18±3.75	76.69±0.15
*D*. *communis*	0:4	50.15±4.35	100.00±0.00	100.00±0.00	41.25±0.77	97.43±3.36	100.00±0.00	56.09±3.41	96.60±0.37	90.00±0.01
	1:3	88.68±0.71	100.00±0.00	100.00±0.00	83.18±3.38	95.36±5.37	100.00±0.00	88.02±0.37	100.00±0.00	99.73±0.00
	2:2	78.05±6.22	100.00±0.00	97.93±0.27	58.24±7.82	88.39±1.49	93.14±0.83	78.12±1.99	80.27±5.90	99.86±5.00
	3:1	75.75±0.84	99.98±0.08	92.53±3.26	51.37±1.14	78.42±0.68	82.30±1.21	76.95±0.58	67.58±4.07	92.88±4.00
	4:0	74.46±2.64	99.42±0.16	89.52±3.03	62.04±1.66	64.97±8.75	81.15±0.46	67.34±0.41	57.01±4.16	82.53±4.16
Blank	0:4	16.96±4.09	10.00±1.38	2.82±7.06	19.47±0.02	26.33±6.13	8.98±7.32	28.25±4.64	17.86±5.75	8.98±3.39
	1:3	11.25±0.97	12.99±0.26	15.81±4.42	19.21±4.68	14.89±2.93	18.98±0.56	19.47±4.30	18.13±1.68	31.66±2.22
	2:2	10.65±0.28	12.21±2.37	17.79±5.95	10.65±0.28	15.03±1.62	22.64±0.91	15.10±1.88	18.14±3.63	25.88±3.67
	3:1	12.62±1.23	5.92±1.49	8.66±2.16	13.67±2.70	8.11±1.62	16.20±6.85	11.68±2.29	14.10±0.33	19.45±3.08
	4:0	12.90±2.87	9.32±0.40	9.11±0.88	10.53±0.49	11.77±3.04	13.98±0.92	10.69±0.26	14.05±4.02	19.67±3.53

When extra CO_2_ was aerated, the removal rate of ammonia showed a decrease in media 2:2, 3:1 and 4:0 (Fig 2c, 2d, 2e, 2f and Table 3). This result was probably due to the dissolution and ionization of CO_2_ and acidification of the media. As shown in Fig 3d, 3e, 3f, 3g, 3h and 3I, the pH value of the media decreased with extra CO_2_ aeration. A low-pH environment could reduce the stripping and loss of ammonia because it promoted the equilibrium concentration of ammonium and suppressed the generation of free ammonia [26, 28]. Thus, although the removal rate of ammonia was decreased under 5% and 10% CO_2_, the ammonia uptake by *C*. *sorokiniana* and *D*. *communis* was not necessarily decreased.

**Fig 4:**
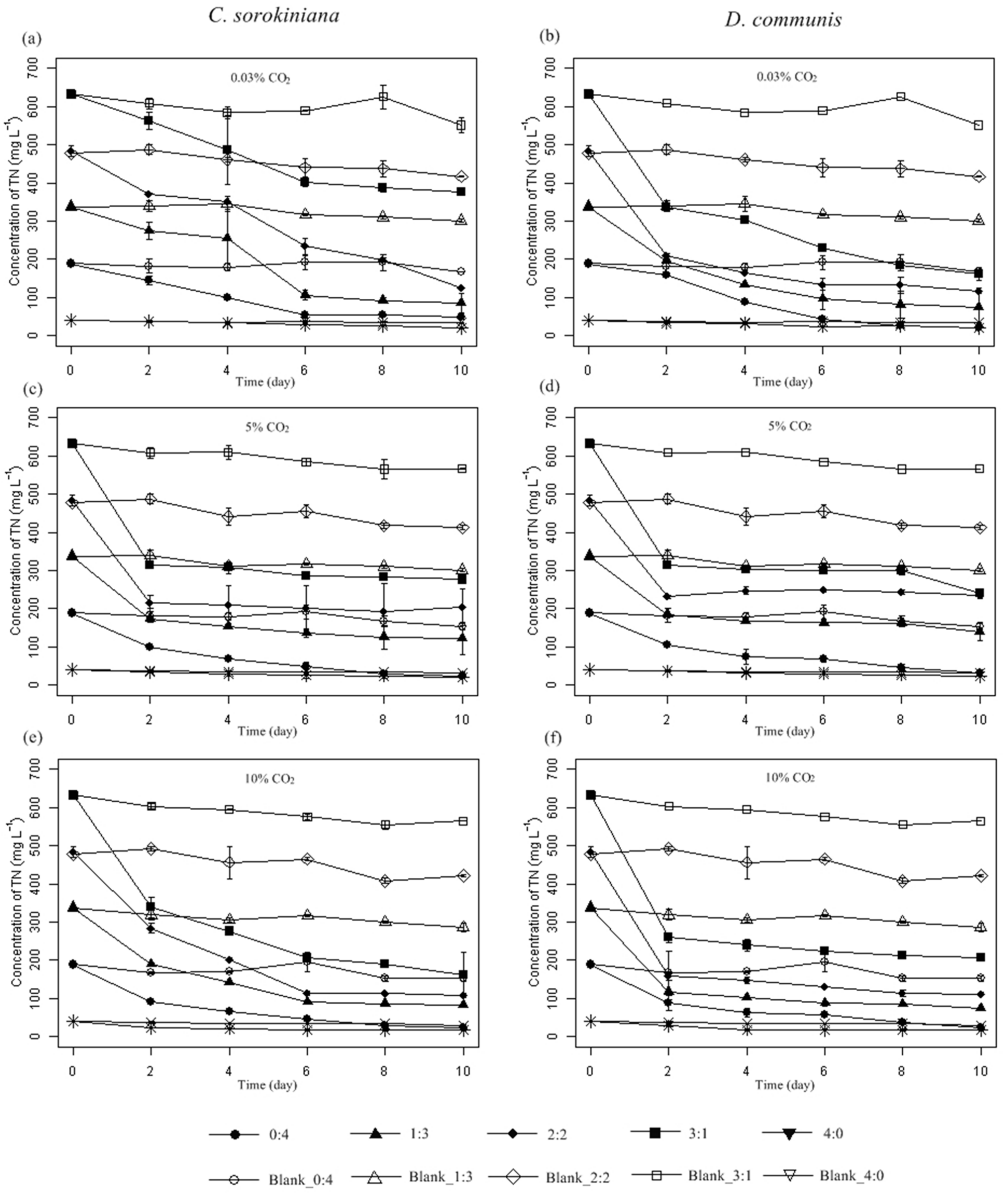
Time course of total nitrogen (TN) evolution for *Chlorella sorokiniana* and *Desmodesmus communis* cultivated in different media under 0.03% (a and b), 5% (c and d) and 10% (e and f) CO_2_ concentrations, respectively. Media without microalgae inoculation was marked as Blank, Error bars represent ±SD of three replicates.

Except for the case of *C*. *sorokiniana* at 0.03% CO_2_, the total nitrogen was reduced to half of the original level after two days for both microalgae (Fig 4). In addition, both *C*. *sorokiniana* and *D*. *communis* reduced more TN in wastewater medium 1:3 than in other media (Table 3). Thus, 1:3 may be an excellent choice for good nutrient-removal capacity and high biomass productivity. The highest TN removal rates achieved by *C*. *sorokiniana* and *D*. *communis* were 88.05% and 88.68%, respectively (Table 3). This result indicated that there were still some organic compounds that could not be assimilated by microalgae, which in consistence with that in a previous report [29].

**Fig 5:**
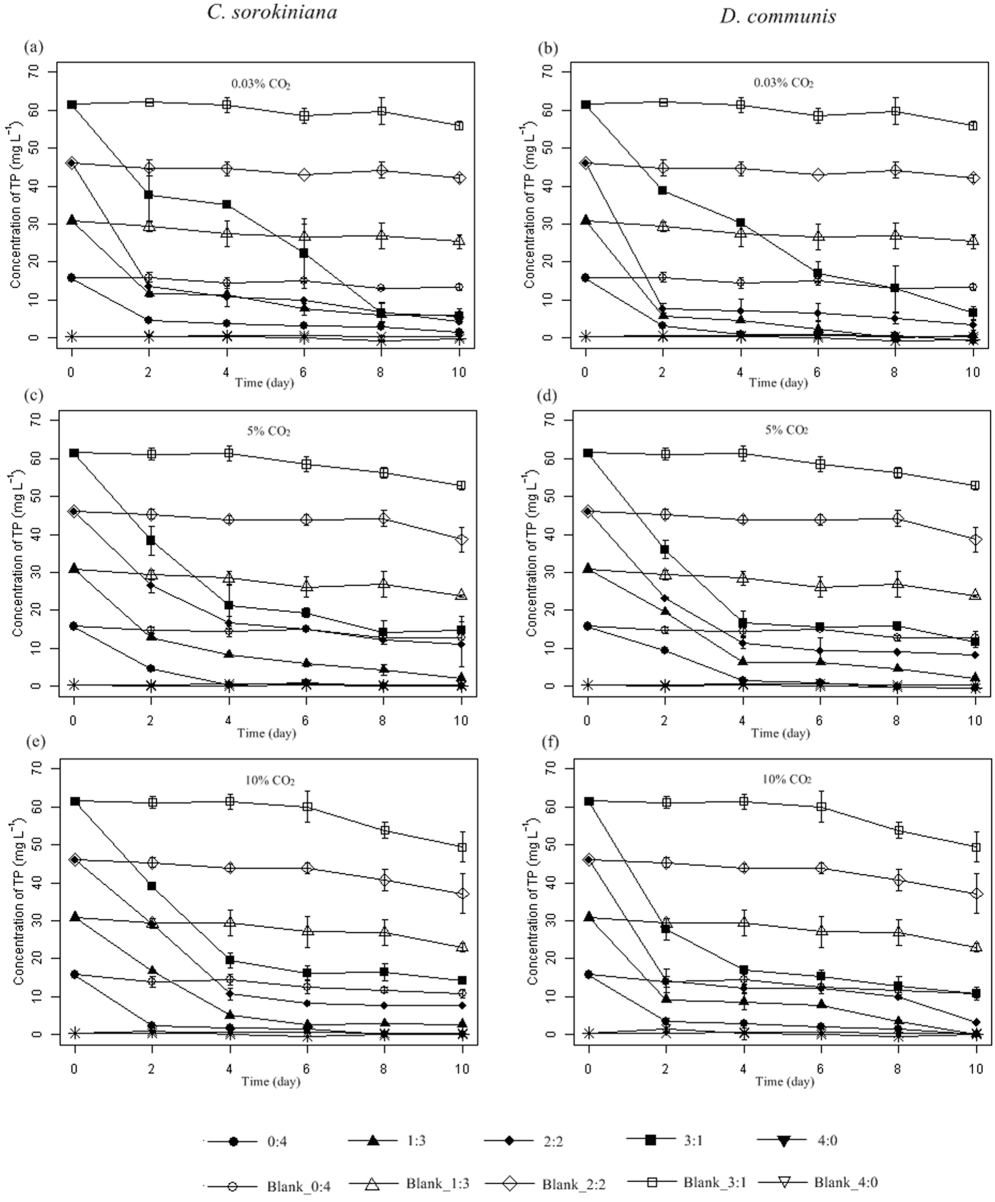
Time course of total phosphorus (TP) evolution for *Chlorella sorokiniana* and *Desmodesmu scommunis* cultivated in different media under 0.03% (a and b), 5% (c and d) and 10% (e and f) CO_2_ concentrations, respectively. Media without microalgae inoculation was marked as Blank, Error bars represent ±SD of three replicates.

Phosphorus can be found in lipids, proteins, nucleic acids and the intermediates of carbohydrate metabolism and is also an essential macro-nutrient for microalgae growth. Fig 5 and Table 3 showed the removal of TP from five wastewater media. It should be noted that the removal of phosphorus in wastewater was not only affected by microalgae cell uptake but also by external conditions such as pH and dissolved oxygen. When the pH is elevated close to 10, phosphate will precipitate from wastewater [30], explaining the high phosphorus removal rate under 0.03% CO_2_ (Fig 5a and 5b and Table 3). In medium 1:3, *C*. *sorokiniana* and *D*. *communis* removed more than 99.5% TP, except for *C*. *sorokiniana* under 0.03% CO_2_ (90.79%). This result showed again that medium 1:3 was suitable for *C*. *sorokiniana* and *D*. *communis* to remove nutrients from wastewater.

When aerated with CO_2_, the mechanisms of nutrient removal by *C*. *sorokiniana* and *D*. *communis* were completely different because the pH variation changed the equilibrium concentration and uptake of nutrients. The pH and initial concentrations of N and P not only affected the growth of *C*. *sorokiniana* and *D*. *communis* but also their removal capacities. In this study, the increase in pH resulted in the high removal rates of nitrogen and phosphorus at 0.03% CO_2_, and medium 1:3 was considered to be the most appropriate for its high removal rates of ammonia, TN and TP.

### Lipid production of microalgae

The lipid contents of *C*. *sorokiniana* and *D*. *communis* cultured in six types of media under 0.03%, 5% and 10% CO_2_ are shown in Table 4. Without additional CO_2_ aeration, *C*. *sorokiniana* and *D*. *communis* had high lipid contents when cultivated in medium 0:4–25.15% and 23.05%, respectively. This result was consistent with those in previous reports that showed that nitrogen limitation would induce incrementally the microalgal lipid content [31–32]. Because lipids are preferred storage compounds that have highly reduced states, they could be efficiently packed in cells and used under stressed conditions for cell survival [31]. When microalgae were cultivated under nutrient-limiting conditions, the photosynthetic carbon flow changes into metabolic pathways that may generate energy-rich compounds, such as lipids[33].

**Table 4:** The lipid content (Lc) and lipid productivity (Lp) of *Chlorella sorokiniana* and *Desmodesmus communis* in different media under 0.03%, 5% and 10% CO_2_ concentrations after10 days culture, respectively. Error bars represent ± SD of three replicates. Since the lipid productivity was calculated as the average value of lipid content multiplied by the average biomass concentration and divided by 10, it is reported as a single value without standard deviation.

	Medium(SW: STMW)	0.03% CO_2_	5% CO_2_	10% CO_2_
		Lc (%)	Lc (%)	Lc (%)
*C*. *sorokiniana*	BG-11	14.15±2.52	17.91±0.74	13.16±0.74
	0:4	25.15±2.13	18.08±2.90	14.94±1.32
	1:3	18.47±0.97	17.04±0.47	10.44±1.11
	2:2	21.89±1.55	17.37±1.86	12.64±3.34
	3:1	19.56±1.80	15.78±1.58	10.09±5.56
	4:0	21.83±2.11	25.10±4.01	15.09±4.06
*D*. *communis*	BG-11	21.51±0.56	22.81±0.23	15.81±5.66
	0:4	23.05±2.80	21.89±2.43	17.28±0.27
	1:3	17.20±0.41	20.37±0.53	14.87±0.56
	2:2	22.21±2.17	22.69±4.66	16.96±2.05
	3:1	23.81±1.21	16.60±2.31	20.65±1.16
	4:0	22.16±1.74	30.33±1.58	22.75±4.01

When 5% and 10% of CO_2_ were aerated, the lipid contents of *C*. *sorokiniana* in medium 4:0 reached 25.10% and 15.09%, respectively, values that were higher than those in other media (Table 4). *D*. *communis* had a similar trend in lipid accumulation to *C*. *sorokiniana*—the lipid content peaked at 30.33% and 22.75% in medium 4:0 under 5% and 10% CO_2_, respectively. These results suggested that nutrient excess could also induce the accumulation of intracellular lipids under one type of environmental stress such as nutrient limitation.

### Morphology change in microalgae during cultivation

**Fig 6:**
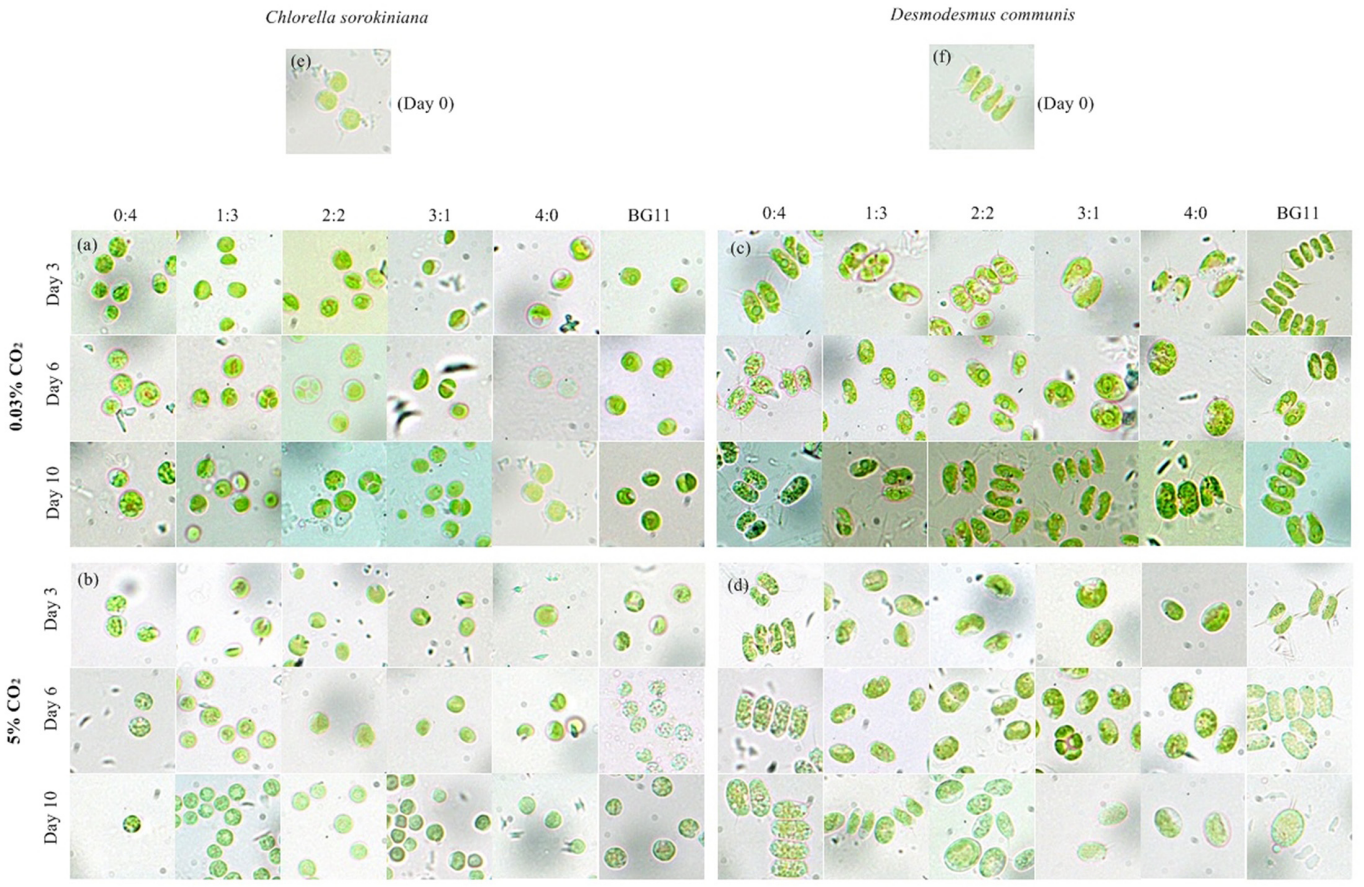
Morphology pictures of *Chlorella sorokiniana* (a and b) and *Desmodesmus communis* (c and d) in different media under 0.03% and 5% CO_2_ concentrations after 3, 6 and 10 days culture, respectively. Pictures of *Chlorella sorokiniana* and *Desmodesmus communis* before inoculation (Day 0) were shown in e and f, respectively.

The morphology of microalgal cells was closely related to their culture conditions, indicating that nutrients and gas aeration could significantly affect the form of microalgal cells [15, 34–35]. The morphological features of *C*. *sorokiniana* and *D*. *communis* cultivated in different wastewater media and modified BG-11 under an aeration of 0.03% and 5% CO_2_ are shown in Fig 6. The morphology of the two microalgae under 10% CO_2_ in photographs was similar with 5%, and images are shown in figure in S1 Fig and will not be discussed here. The cells of *C*. *sorokiniana* cultivated in medium 0:4 generated more intracellular particles than cells in other media after 3 days of culture under 0.03% CO_2_ (Fig 6a). When microalgae were cultivated in medium 0:4, a nitrogen limitation condition in this study, photosynthetic carbon flow changes into metabolic mechanisms that may generate energy-rich compounds, such as carbohydrates and lipids [33]. When aerated with CO_2_, both 5% and 10%, a significantly increase of intracellular particles number in *C*. *sorokiniana* cells cultivated in medium 1:3 and BG-11 was observed by an optical microscope after 6 days of cultivation (Fig 6b).

The morphological changes in *D*. *communis* had a similar tendency to *C*. *sorokiniana*. In addition, *D*. *communis* has other features because it is a strain belonging to the *Scenedesmaceae* family. The cells are displayed as a 4-celled cenobium before inoculation (Fig 6f). However, we can only observed dispersed solitary cells when *D*. *communis* was cultivated in media 1:3, 2:2, 3:1 and 4:0 on the 6^th^ day under a 0.03% CO_2_ concentration (Fig 6c). However, on the 10^th^day, most cells were grouped into 2- or 4-celled cenobium again and only a small number of cells remained solitary (Fig 6c). This phenomenon was also found in *Scenedesmus* sp. CCNM 1077 [15]. When additional CO_2_ was aerated, it appeared earlier, and no 2- or 4-celled cenobium could be found on the 3^rd^ day (Fig 6d). Solitary cells showed a remarkable morphological difference compared with cenobium. They presented with a more regular ellipsoid with a larger size, and the spines became unobtrusive. *D*. *communis* cells all remained solitary except in media 0:4 and 1:3 under 5% CO_2_ (Fig 6d), a finding that was different from that under 0.03% CO_2_. Combined with the result of growth (Fig 1), we hypothesized that 4-celled cenobium was not conducive to cell division and growth because *D*. *communis* tended to be solitary or 2-celled during the logarithmic phase. Because CO_2_ aeration promoted growth, *D*. *communis* had a stronger tendency to be solitary.

## Conclusions

In summary, the present study showed that it is feasible to increase biomass and total lipid productivity by mixing SW and STMW coupled with a proper CO_2_ concentration. Both *Chlorella sorokiniana* and *Desmodesmus communis* cultivated in 1:3 (SW:STMW) medium achieved the highest nutrient removal rate with or without extra CO_2_ aeration. *C*. *sorokiniana* obtained the maximum biomass concentration (1.31g L^-1^) and maximum lipid productivity (0.023g L^-1^ d^-1^) in medium 2:2 (SW:STMW) under 5% CO_2_ concentration. The results suggested that SW and STMW have great potential to become sources of nutrition for microalgae by mixing them at a suitable ratio.

## Supporting Information

S1 Fig Morphology pictures of *Chlorella sorokiniana* (a) and *Desmodesmus communis* (b) in different media under 10% CO_2_ concentrations after 3, 6 and 10 days culture, respectively. Pictures of *Chlorella sorokiniana* and *Desmodesmus communis* before inoculation (Day 0) were shown in c and d, respectively.
(DOCX)
