# Supplementary material for: Mixed Wastewater Coupled with CO2 for Microalgae Culturing and Nutrient Removal
Source: PLoS One. 2015 Sep 29;10(9):e0139117. doi: 10.1371/journal.pone.0139117 (PMC4587883; doi:10.1371/journal.pone.0139117)

**S1 Fig. Morphology pictures of *Chlorella sorokiniana* (a) and *Desmodesmus communis* (b) in different media under 10% CO_2_ concentrations after 3, 6 and 10 days culture, respectively.** Pictures of *Chlorella sorokiniana* and *Desmodesmus communis* before inoculation (Day 0) were shown in c and d, respectively.


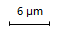

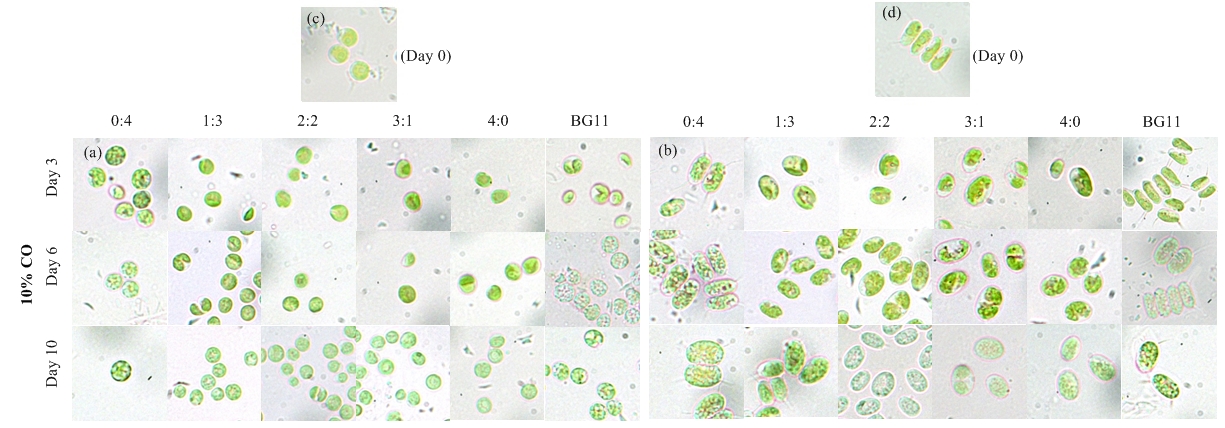

Supplement: S1 Fig — Pictures of Chlorella sorokiniana and Desmodesmus communis before inoculation (Day 0) were shown in c and d, respectively. (DOCX) [file pone.0139117.s001.docx]
